# GIP as a Potential Therapeutic Target for Atherosclerotic Cardiovascular Disease–A Systematic Review

**DOI:** 10.3390/ijms21041509

**Published:** 2020-02-22

**Authors:** Yusaku Mori, Takanori Matsui, Tsutomu Hirano, Sho-ichi Yamagishi

**Affiliations:** 1 Division of Diabetes, Metabolism, and Endocrinology, Department of Medicine, Showa University School of Medicine, Shinagawa, 142-8555 Tokyo, Japan; hirano@med.showa-u.ac.jp (T.H.); shoichi@med.showa-u.ac.jp (S.-i.Y.); 2 Department of Pathophysiology and Therapeutics of Diabetic Vascular Complications, Kurume University School of Medicine, Kurume, 830-0011 Fukuoka, Japan; matsui_takanori@med.kurume-u.ac.jp; 3 Diabetes Center, Ebina General Hospital, Ebina, 243-0433 Kanagawa, Japan

**Keywords:** animal model, atherosclerosis, cardiac remodeling, GIP, restenosis

## Abstract

Glucose-dependent insulinotropic polypeptide (GIP) and glucagon-like peptide-1 (GLP-1) are gut hormones that are secreted from enteroendocrine L cells and K cells in response to digested nutrients, respectively. They are also referred to incretin for their ability to stimulate insulin secretion from pancreatic beta cells in a glucose-dependent manner. Furthermore, GLP-1 exerts anorexic effects via its actions in the central nervous system. Since native incretin is rapidly inactivated by dipeptidyl peptidase-4 (DPP-4), DPP-resistant GLP-1 receptor agonists (GLP-1RAs), and DPP-4 inhibitors are currently used for the treatment of type 2 diabetes as incretin-based therapy. These new-class agents have superiority to classical oral hypoglycemic agents such as sulfonylureas because of their low risks for hypoglycemia and body weight gain. In addition, a number of preclinical studies have shown the cardioprotective properties of incretin-based therapy, whose findings are further supported by several randomized clinical trials. Indeed, GLP-1RA has been significantly shown to reduce the risk of cardiovascular and renal events in patients with type 2 diabetes. However, the role of GIP in cardiovascular disease remains to be elucidated. Recently, pharmacological doses of GIP receptor agonists (GIPRAs) have been found to exert anti-obesity effects in animal models. These observations suggest that combination therapy of GLP-1R and GIPR may induce superior metabolic and anti-diabetic effects compared with each agonist individually. Clinical trials with GLP-1R/GIPR dual agonists are ongoing in diabetic patients. Therefore, in this review, we summarize the cardiovascular effects of GIP and GIPRAs in cell culture systems, animal models, and humans.

## 1. Introduction

Atherosclerotic cardiovascular disease (CVD) is a major cause of death and disability among individuals with diabetes in many countries [1,2]. Indeed, a hazard ratio among individuals with diabetes as compared with those without diabetes was 1.8 for death from CVD even after adjusting for several well-known risk factors such as high systolic blood pressure and serum cholesterol levels [1]. However, the effects of strict blood glucose control on CVD are marginal, especially in diabetic patients with a long disease history [3,4,5,6,7]. These observations suggest that development of novel therapeutic strategies is needed to further reduce the risk of CVD and subsequently improve the quality of life in both type 1 and type 2 diabetic patients [1,8,9,10].

Glucose-dependent insulinotropic polypeptide (GIP) and glucagon-like peptide-1 (GLP-1) are gut hormones that are secreted from enteroendocrine L cells and K cells, respectively, in response to stimuli with digested nutrients [11,12]. GIP and GLP-1 are also referred to as incretins for their ability to stimulate insulin secretion from pancreatic beta cells in a glucose-dependent manner. Furthermore, GLP-1 exerts anorexic effects through its actions on the central nervous system [11,12]. Since native incretin is rapidly inactivated by dipeptidyl peptidase-4 (DPP-4), DPP-resistant GLP-1 receptor agonists (GLP-1RAs) and DPP-4 inhibitors have been developed and now widely used for the treatment of diabetic patients as incretin-based agents because of their superiority to classical insulinotropic agents, such as sulfonylureas and glinides from the standpoint of low risks for hypoglycemia and body weight gain [13,14,15].

Both GIP and GLP-1 have been reported to exert direct effects on the cardiovascular system in addition to pancreatic beta cells [12,16]. Indeed, a number of preclinical studies, including ours, have shown the cardiovascular protective effects of native incretins and incretin-based agents partly in a glucose-lowering independent manner [17,18,19,20,21,22,23,24,25,26,27,28], whose observations were consistent with the recent cardiovascular outcome trials demonstrating that GLP-1RAs significantly reduced the cardiovascular and renal events in high-risk type 2 diabetic patients compared with placebo [29,30,31,32,33]. Furthermore, DPP-4 inhibitors have also been shown to improve surrogate markers of atherosclerotic CVD in several clinical trials [34,35,36], although their effects on CV hard events were neutral in various cardiovascular outcome trials [37,38,39,40,41]. Based on these clinical findings, GLP-1RAs have now recommended as one of the first-line therapies in metformin-treated type 2 diabetic patients with high risks or established CVD. 

In contrast to the case of GLP-1, no GIP receptor (GIPR) agonist is clinically utilized to date because its therapeutic potential was doubted by the observations showing impaired insulinotropic effects of GIP in individuals with diabetes [42,43,44]. In addition, inhibition of physiological GIP has been shown to prevent the body weight gain in high-fat diet (HFD) fed mice by suppressing the GIP-induced adipogenesis in adipose tissues [45,46,47,48], thereby raising safety concerns that GIP treatment could promote obesity and deteriorate the metabolic risks in type 2 diabetic patients. Therefore, cardiovascular effects of GIP had been almost neglected. However, paradigm of GIP-based therapy has been changed by recent preclinical studies, which showed that administration of GIP analogs at pharmacological doses or overexpression of GIP could suppress the HFD-induced body weight gain as is the case of the inhibition of physiological GIP, probably through the anorexic effects of GIP on the central nervous system [49,50,51,52,53]. These observations were further supported by a recent clinical trial, which showed that LY3298176, a dual agonist targeting for GIPR and GLP-1R exerted superior effects on glycemic controls and body weight reductions compared with GLP-1RA, dulaglutide monotherapy in patients with type 2 diabetes [54]. Given the fact that clinical trials with dual (GLP-1R/GIPR) or triple (GLP-1R/GIPR/glucagon receptor) agonists are ongoing in diabetic patients [55,56], attention will be paid whether combination therapy with incretin-based therapy, such as GIPR agonists and GLP-1RAs, could be more effective for preventing the CVD than GLP-1RA monotherapy in type 2 diabetic patients. However, to data, there is no clinical trial to address the issue. Therefore, this article summarizes the cardiovascular effects of GIP and GIPRAs in cell culture systems, animal models, and humans. Although several observational studies reported the correlation between circulating GIP levels and presence or severity of atherosclerotic CVDs, these data are not included in this review because we cannot draw a definite conclusion from observational studies whether GIP acts as a causal or compensative protective factor for CVD. In this review, literature searches were undertaken in Medline by the PubMed interface. Non-English language articles were excluded. Key words (GIP [glucose-dependent insulinotropic polypeptide, glucose-dependent insulinotropic peptide, or gastric inhibitory polypeptide] and (atherosclerosis, restenosis, heart, artery, inflammation, adipose tissue, endothelial cell, smooth muscle cell, monocyte, macrophage, adipocyte, and desensitization) have been used to select the articles.

## 2. Cell Culture Studies

### 2.1. Vascular Endothelial Cells (VECs) 

VEC plays a central role in the maintenance of cardiovascular homeostasis mainly via nitric oxide (NO) production [57], and their dysfunction is involved in the pathogenesis of early phase of atherosclerosis [58]. GIPR has also shown to be expressed in various types of VECs [27,59,60,61,62,63]. GIPR belongs to the glucagon receptor subfamily of G protein-coupled receptor class B, and its signaling is mainly mediated by cyclic adenosine monophosphate (cAMP) pathway in pancreatic beta cells [11,12]. However, GIPR signaling pathway in VECs is possibly distinct from that in pancreatic beta cells, whereas effects of GIP may differ among VEC types [60,64,65,66]. There is an in vitro study showing the different effects of GIP on VECs isolated from hepatic artery and portal vein of canine [64]. GIP stimulated cell proliferation in either type of VECs without affecting intracellular cAMP levels. However, ranging from 0.1 to 10 nM, GIP dose-dependently increased the intracellular calcium levels and NO production without affecting endothelin-1 production in ECs of portal vein. On the other hand, GIP did not affect the intracellular calcium levels or NO production, but increased endothelin-1 production in ECs of hepatic artery. These observations are consistent with in vivo findings that GIP infusion dose-dependently increased portal vein blood flow but decreased hepatic artery blood flow in conscious dogs [65]. The subsequent study by the same group showed that immortalized ECV 304 cells and ECs collected from human umbilical vein (HUV), aorta, and pulmonary artery contained different splicing patterns of GIPR [66]. Indeed, responses to GIP stimulation, as assessed with elevations in intracellular calcium levels, were different among these VECs, and GIP-induced activation of cAMP-dependent protein kinase, also known as protein kinase A (PKA), was observed only in HUVEC. These observations were further supported by a study that ranging from 0.1 to 100 nM, GIP concentration-dependently increased the endothelin-1 (ET-1) production in HUVECs, but not ECV 304 cells [60]. Although it remains unclear why effects of GIP are varied between VEC types, a couple of studies suggested the involvement of GIPR splicing variants in GIP actions [67,68]. The truncated GIPR retaining intron 8 was co-expressed with functional GIPR in pancreatic beta cells of mice, and the induction of truncated GIPR gene impaired the GIP-induced cAMP production in cells expressing functional GIPR [67]. Furthermore, 64 possible variants of GIPR were detected in human adipose tissues, and only two of them contained the functional domain [68]. However, further studies are needed to clarify whether GIPR splicing variants can be involved in altered GIP actions between VEC types. 

Anti-atherogenic effects of GIP on VECs were reported by several studies using HUVECs (Table 1) [27,61,63]. Accumulation of advanced glycation end products (AGEs) is a causal factor for atherosclerosis through dysfunction, inflammation, apoptosis, and other various adverse responses of VECs [69,70]. We have previously found that active GIP at 50 pM inhibited the generation of reactive oxygen species via reductions in gene expression levels of *receptor for AGEs* in AGEs-exposed HUVECs, with concomitant reductions in gene expression levels of pro-atherogenic molecules, such as *vascular cell adhesion molecule* and *plasminogen activator inhibitor-1* (*Pai-1*) [61]. GIP at 1 nM also increased the production of NO, the potent anti-atherogenic molecule [57] through the activation of endothelial nitric oxide synthase (NOS) in HUVECs, whereas it decreased inflammatory inducible NOS expression levels [63]. Furthermore, we have found that active, but not inactive GIP at 1000 nM increased the NO production via the activation of AMP-activated protein kinase (AMPK) in HUVECs [27], while GIP-induced AMPK activation was mediated by phospholipase C (PLC) and calcium/calmodulin-dependent protein kinase kinase (CaMKK), but not adenyl cyclase or liver kinase B1 (LKB1). These findings suggest the possible involvement of GIPR/PLC/CaMKK/AMPK/NO axis in the anti-atherogenic effects of GIP [71,72,73]. However, a couple of studies reported the pro-atherogenic effects of GIP on VECs (Table 1) [60,62,64]. As above-mentioned, GIP increased the production of ET-1 [60,64], which acts as a pro-atherogenic molecule [74]. Another study showed that GIP evoked the ET-1 production in aortic ECs of mice via cAMP response element-binding protein (CREB)-dependent, but cAMP/PKA-independent mechanisms [62]. In addition, EC-produced ET-1 subsequently stimulated the pro-atherogenic osteopontin production in aortic smooth muscle cells (SMCs) [62,75]. In consistent with the observations, GIP-induced elevations in ET-1 and osteopontin protein productions were observed in isolated mouse aorta ex vivo [62]. However, it remains unclear whether the inhibition of physiological levels of GIP can result in the suppression of atherosclerosis in vivo. Taken together, GIP could exert both anti-atherogenic and pro-atherogenic effects, which may depend on VEC types. 

### 2.2. Vascular Smooth Muscle Cells (VSMCs)

VSMCs exist in the media of vasculatures as contractile form under physiological conditions. Growth factors, some of which can be produced from atherosclerotic plaque, induce a phenotypic switch from contractile to synthetic form, which are prone to proliferation, migration and extracellular matrix production, thereby contributing to the progression of atherosclerosis [85]. GIPR protein was barely detected in the media of mouse aorta [62]. However, gene expression levels of Gipr in cultured mouse aortic SMCs were upregulated by stimulation with growth factors, thus suggesting that GIP may act on synthetic, but not contractile form of VSMCs [62]. Indeed, we have found that active GIP suppressed the growth-factor-stimulated cell proliferation in human aortic SMCs (Table 1) [19]. On the other hand, GIP did not directly affect osteopontin production, which was indirectly evoked by GIP-induced production of ET-1 from VECs [62]. However, the underlying mechanisms and other biological effects of GIP on VSMCs remain to be elucidated. 

### 2.3. Monocytes, Macrophages, and Adipocytes

The cascade of monocyte attachment and infiltration to vessel walls, differentiation to macrophages, and form cell formation contribute to the pathogenesis of atherosclerosis [85,86,87,88,89,90,91]. In addition, adipose tissue inflammation also plays a role in the promotion of atherosclerosis through the altered production of inflammatory cytokines/chemokines and adipokines [92]. We have previously found that GIPR is expressed in monocytes, but its level is considerably downregulated after differentiation to macrophages, thereby indicating the involvement of GIP in the inflammatory responses [19]. Indeed, a couple of studies showed the anti-inflammatory effects of GIP in immune cells (Table 1) [76,77]. GIP ranging from 1 to 100 nM dose-dependently suppressed the lipopolysaccharide (LPS)-induced gene expression levels of *tumor necrosis factor* (*TNF*) or *inducible NOS* in human monocyte THP-1 cells, and these effects were abolished by inhibiting adenyl cyclase or PKA, but not exchange protein directly activated by cAMP 2 [76]. This suggests the involvement of GIPR/adenyl cyclase/PKA axis. The anti-inflammatory effects of GIP on monocytes were further demonstrated by a recent study showing that active GIP suppressed the chemokine ligand 2 (CCL2)-induced migration of mouse monocyte RAW 264 cells and human monocyte THP-1 cells thorough reductions in gene expression levels of *chemokine receptor type 2* [77]. In addition, active GIP also inhibited the matrix metalloproteinase-9 protein levels and interleukin (IL)-6 production by suppressing LPS-induced activation of nuclear factor-kappa B p65 and mitogen-activated protein kinases in mouse monocyte RAW 264 cells [77]. 

GIPR has also been shown to be expressed abundantly in differentiated, but not undifferentiated premature adipocytes [82,93,94], and its expression levels were also upregulated by various stimuli, such as IL-1β or hypoxia-inducible factor-1 α activator, and exposure to hypoxic conditions [82]. Anti-inflammatory effects of GIP on adipocytes are reported by several studies (Table 1) [79,80]. GIP at 100 nM increased the gene expression levels of Adiponectin in isolated rat and human adipocytes [79]. Furthermore, DPP4-resistant [D-Ala2] GIP at 100 nM in the presence of insulin reduced the gene expression levels of *IL-1 beta*, *IL-6*, *CCL8*, and *progranulin* in adipocytes collected from mesenteric adipose tissue of obese individuals [80]. However, pro-inflammatory effects of GIP on adipocytes are also reported by several studies (Table 1) [68,78,81,82,83,84]. In differentiated mouse 3T3-L1 adipocytes overexpressing GIPR, GIP increased the gene expression levels of *Il-6*, *Tnf-alpha*, *Ccl2* and *Ccl7*, but decreased those of *adiponectin* and *leptin*, which were mediated by IKKβ or PKA, and partially by c-Jun-NH2-terminal kinase [84]. In addition, subsequent studies reported that GIP at 1 or 100 nM also increased the protein levels of IL6 and IL-1 receptor antagonist in the presence of LPS, IL-1β, or TNF-α in adipocytes obtained from subcutaneous abdominal fat pads of non-obese and obese individuals [81], and that GIP at 10 nM increased the production of IL6 and CCL2 in differentiated mouse 3T3-L1 adipocytes [82]. Another study reported that GIP at 100 nM increased the gene expression levels of *Ccl2* in mouse monocyte RAW 264 cells, but not differentiated mouse 3T3-L1 adipocytes [78]. GIP at 1 to 100 nM also increased the protein levels of osteopontin in insulin-treated mouse 3T3-L1 adipocytes and isolated rat adipocytes via the transcription factor nuclear factor of activated T-cells [83], which was further confirmed in cultured rat visceral adipocytes under normal and high glucose conditions [68]. Collectively, GIP is likely to exert anti-inflammatory effects on monocytes, whereas its effects on adipocyte inflammation are controversial.

## 3. Animal Studies

### 3.1. Atherosclerosis Models

Atherosclerosis is the main cause of tissue ischemia and infarction in brains and hearts, and its prevention has long been a potential therapeutic target to suppress the CVD in individuals with diabetes [1,2]. Anti-atherogenic effects of GIP in mouse models were reported by several research groups (Table 2) [19,21,77]. We have found in atherosclerosis-prone apolipoprotein E knockout (*ApoE-/-*) mice [95] that chronic infusion of active GIP (25 nmol/kg/day) for 4 weeks suppresses the aortic plaque formation and intra-plaque macrophage accumulation compared with vehicle treatment, whose effects were totally independent of food intake, body weight, systolic blood pressure, and plasma glucose and lipid levels [19]. In our study, inactive GIP infusion showed no effects on atherosclerosis. Furthermore, active GIP, but not inactive GIP infusion, suppressed the macrophage foam cell formation in ex vivo experiments, which plays a crucial role in the development and progression of atherosclerosis [90,91], whereas it decreased protein expression levels of CD36 and acetyl-coenzyme A acetyltransferase-1, which are involved in oxidized low density lipoprotein (LDL) uptake and intracellular cholesterol storage, respectively. We have also observed the anti-atherogenic effects of GIP in diabetic animals [21]. The infusion of active GIP (25 nmol/kg/day) for 4 weeks reduced the aortic plaque formation, intra-plaque macrophage accumulation, macrophage foam cell formation in streptozotocin-induced diabetic *ApoE-/-* mice. In addition, recently, overexpression of GIP has been reported to stabilize the atherosclerotic plaque in non-diabetic *ApoE-/-* mice [77]; overexpression of GIP gene reduced macrophage accumulation and increased collagen content of aortic plaques, while it did not induce body weight gain. Taken together, these findings suggest that GIP at pharmacological concentrations could exert protective effects against atherosclerosis without deteriorating the obesity, and the beneficial effects of GIP might also be preserved in diabetic animals. However, it remains unclear whether GIP infusion at the physiological dose suppresses or promotes atherosclerosis in animal models. 

### 3.2. Restenosis Models

Percutaneous transluminal angioplasty (PTA) and bypass graft are common revascularization procedures for the treatment of atherosclerotic vascular disease that could cause the ischemia or infarction of hearts [96,97]. PTA in combination with drug-eluting stents can be widely applicable to coronary heart disease patients with various comorbidity due to its less invasiveness than bypass graft. However, its long-term coronary artery patency is still limited by substantial rate of restenosis, especially in high-risk patients, such as diabetic subjects [96,97]. One of the main causes of restenosis is neointimal hyperplasia, which chronically occurs at the site of the intervention as an exaggerated healing response to vascular injury caused by PTA procedures [98]. We have previously investigated the effects of pharmacological and physiological doses of GIP on restenosis using mouse models of femoral artery wire injury (Table 2) [27]. The infusion of active GIP (50 nmol/kg/day), but not inactive GIP for 4 weeks suppressed the injury-induced neointimal hyperplasia and vascular cell proliferation without affecting the metabolic parameters, including body weight gain. In addition, active GIP infusion also facilitated the regeneration of VECs, which were completely denuded after wire insertion [27]. Furthermore, the active GIP infusion also significantly reduced the injury-induced neointimal hyperplasia and vascular cell proliferation in diabetic *db/db* mice, an animal model of type 2 diabetes with obesity as well [27]. Interestingly, co-treatment with a NOS inhibitor, nitro-L-arginine methyl ester completely abolished the beneficial effects of active GIP, thus suggesting the involvement of NO-dependent mechanisms in atheroprotective actions of GIP [27]. In contrast, whole-body deletion of GIPR gene in wild-type mice promoted the neointimal hyperplasia after vascular injury [27]. Therefore, pharmacological GIP may be beneficial to inhibit the restenosis after PTA through the suppression of neointimal hyperplasia both in non-diabetic and diabetic animals.

### 3.3. Cardiac Remodeling Models

Heart failure has become one of the major causes of CV death in individuals with diabetes, who are at high risk for heart failure and death [102,103]. The pathology of heart failure in diabetes is associated with cardiac remodeling caused by complexes of atherosclerotic coronary disease and diabetic cardiomyopathy, resulting in systolic and diastolic dysfunction [104,105,106]. However, effective therapy to ameliorate cardiac remodeling in diabetes is still limited. GIPR has been shown to be expressed both in the atrium and ventricle of mice and human hearts, thus suggesting that the heart is one of the extra-pancreatic target organs for GIP [25,59,99]. We have previously found that GIP plays a protective role against cardiac remodeling in *ApoE-/-* mice infused with angiotensin II (Table 2) [25], whose pathway is also activated in diabetic cardiomyopathy [104,105,106]. Indeed, infusion of active GIP (25 nmol/kg/day) for 4 weeks suppressed the left ventricle cardiomyocyte enlargement and interstitial fibrosis, which were associated with concomitant reductions in cell apoptosis and transforming growth factor-β protein expression in the hearts of angiotensin II-infused *ApoE-/-* mice [25]. In other studies, effects of GLP-1 on infarcted rat hearts were evaluated [99,107,108,109]. In the infarcted rat hearts induced by coronary artery ligation, perfusion with GIP, but not GLP-1 at the infusion rate at 100 nmol/l for 1 h reduced the protein expression levels of resistin [107], a promoter of cardiac remodeling and dysfunction [108,109]. In contrast to the findings, deleterious effects of GIP on cardiac remodeling were also reported in mouse models of myocardial infarction (Table 2) [99]. GIPR gene expression levels in the heart were upregulated at one day but recovered to the baseline at two days after the induction of myocardial infarction. One-week pre-treatment with [D-Ala2] GIP injections (24 nmol/kg body weight, twice daily), did not affect the mortality, but increased the left ventricle scar formation at two weeks after the induction of myocardial infarction in wild-type mice. On the other hand, both whole-body and cardiomyocyte-specific deletion of GIPR gene reduced the infarction-induced mortality, ventricular weight, and left ventricle scar formation [99]. Interestingly, whole-body deletion of GIPR gene did not affect the survival or left ventricle function in doxorubicin-induced or transverse aortic-constriction-induced heart failure mice [99]. Collectively, these findings suggest that effects of GIP on cardiac remodeling may differ depending on experimental conditions. Administration of GIP at the pharmacological dose may prevent the angiotensin II-induced cardiac hypertrophy, while inhibition of physiological levels of GIP could suppress the cardiac remodeling after myocardial infarction. 

### 3.4. Inflammation Models

Inflammation plays a crucial role in the development and progression of atherosclerotic CVD [85,86,87,88,89]. Several studies have shown the anti-inflammatory properties of GIP (Table 2) [49,50,79,80]. The injections of [D-Ala2] GIP (0.12 mg/kg body weight, once daily) decreased the adipose tissue gene expression levels of pro-inflammatory cytokines, such as *Il-1beta*, *Il-6*, *Tnf-alpha* in wild-type mice fed with standard diet [49]. Moreover, overexpression of GIP gene attenuated the gene expression levels of pro-inflammatory molecules, including *Ccl2*, *inhibitor of nuclear factor kappa-B kinase subunit beta*, *Il-4 receptor alpha*, *Tnf receptor superfamily member 1b*, and *Pai-1* in the epididymal fats of HFD-fed mice [50]. Consistent with the case of GIP overexpression, administration of GIP (10 μg/kg, twice daily) for 2 weeks increased the blood levels of adiponectin, an anti-inflammatory and insulin-sensitizing adipokine in HFD-fed rats, which were accompanied with its increased gene expression and decreased *Tnf-alpha* and *Pai-1* gene in the stromal vascular fraction of epididymal fat [79]. In addition, once daily injections of [D-Ala2] GIP (0.12 μg/g) decreased the infiltration of inflammatory immune cells into, and gene expression levels of *Tnf-alpha*, *Il-1beta*, *interferon-γ*, *fractalkine*, *Ccl2*, *Ccl5*, and *Ccl8* in, the epididymal fat of HFD-fed mice, which were accompanied with reductions in the number of circulating bone marrow-derived monocytes and neutrophils [80]. On the other hand, the [D-Ala2] GIP injections increased the epididymal gene expression and blood levels of adiponectin [80]. The anti-inflammatory effects of GIP have also been observed in other tissues [76,101]. Whole-body deletion of GIPR gene promoted the gingival macrophage infiltration and gene expression levels of *Tnf-alpha* and *inducible nos* in a mouse model of periodontitis [76]. The infusion of active GIP (4 pmol/kg/min) reduced the blood levels of IL-6 in LPS-induced endotoxemic mice as well [101]. However, opposite results were obtained in a couple of studies (Table 2) [82,100]. Twice daily injections of GIP (5 nmol/kg) for 1 week augmented the gene expression levels of *Ccl2* and *Il-6* in the retroperitoneal or perirenal fat of *db/db* mice, and also increased the *Ccl2* gene expression levels and macrophage infiltration in the retroperitoneal fat of non-diabetic lean mice [82]. In another study, adipocyte-specific deletion of GIPR gene in HFD-fed mice decreased the lean mass weight and liver steatosis, but not visceral or subcutaneous fat weight, with concomitant reductions in blood levels of IL-6 and adipose tissue gene expression levels of *Il-6* and *suppressor of cytokine signaling 3*, which is a downstream mediator of IL-6 [100]. Taken together, physiological levels of GIP may promote adipose tissue inflammation, whereas GIP at the pharmacological dose could exert anti-inflammatory effects in the adipose tissues.

### 3.5. Limitation of Animal Studies

Male animals were exclusively used in the animal studies (Table 2), possibly due to avoiding potential CV protective effects of estrogen, which needs to be considered in the case of female animals. However, diabetes confers a higher relative risk of CVD mortality among women than among men [110,111]. It remains completely unclear whether the CV effects of GIP demonstrated in male animals can be observed in female non-diabetic and diabetic mice.

## 4. Human Studies

### 4.1. Blood Flow and Blood Pressure

The effects of GIP on blood flow and blood pressure are reported by a couple of studies, in which GIP was intravenously infused at physiological doses that could mimic post-prandial blood levels of GIP (Table 3) [112,113]. GIP infusion, which was started at 10.8 pmol/kg/min and gradually decreased to 4.0 pmol/kg/min at 240 min, reduced the mean arterial blood pressure by 10–15 mmHg, and increased the heart rate by < 8 bpm in individuals with normal glucose tolerance, impaired glucose tolerance, or type 2 diabetes (n = 10 [men, 6; women, 4], 10 [men, 6; women, 4], and 9 [men, 3; women, 6], respectively; no information for ethnicity) [114]. Another study investigated the effects of GIP on blood flow in non-obese young healthy men (*n* =10, no information for ethnicity) under the pancreatic clamp, which was composed of the co-infusion of somatostatin, insulin, glucagon, and growth hormone at the fixed dose of each [115]. GIP infusion at 1.5 pmol/kg/min for 1.5 h increased the blood flow in the femoral artery, but not those in brachial or carotid artery or flow-mediated brachial artery dilation in the hyperglycemic phase, whereas the same infusion of GIP showed no effect on these parameters in the normoglycemic phase. GIP-induced reductions in systolic blood pressure and increases in arterial blood flow could play a protective role against CVD, whereas GIP-induced increases in heart rate may have harmful effects. Given the fact that CV events were reduced by treatment with GLP-1RAs, which could induce similar changes in systolic blood pressure and heart rate as GIP [29,30,31,32,33], overall effects of GIP may be beneficial against CVD.

### 4.2. Inflammatory Cytokine and Chemokine

There are a couple of studies evaluating the effects of GIP on pro-inflammatory cytokines in humans (Table 3) [62,78]. GIP infusion at 2 pmol/kg/min for 240 min increased the blood levels of CCL2 or CCL8 and subcutaneous adipose tissue gene expression levels of *CCL2*, *CCL8*, and *IL-6* in obese men with normal glucose tolerance (*n* = 17, no information for ethnicity) [78]. In addition, these changes were also observed both under euglycemic- and hyperglycemic-hyperinsulinemic clamps. In another study enrolling healthy subjects (*n* = 47, no information for gender and ethnicity), GIP infusion at 4 pmol/kg/min for 105 min increased the blood levels of osteopontin, which was dependent on GIPR genotypes under the hyperglycemic clamp [62]. These observations are consistent with the in vivo findings that physiological level of GIP is involved in adipose tissue inflammation in animal models [82,100]. However, it remains unclear whether pharmacological concentrations of GIP can induce similar pro-inflammatory changes, or conversely suppress the inflammation because both physiological GIP inhibition and pharmacological GIP administration can similarly induce anti-obesity effects [46,47,48,49,50,52]. In addition, data is still missing to elucidate roles of gender and ethnicity in effects of GIP on inflammation.

## 5. Concerns about GIP Therapy

### 5.1. GIPR Downregulation under Hyperglycemia

As above-mentioned, insulinotropic effects of GIP has been shown to be impaired in individuals with diabetes, possibly through hyperglycemia-induced downregulation of GIPR expression in pancreatic beta cells [42,43,44,116,117,118]. However, influence of hyperglycemia on GIPR expression in vascular cells is controversial [21,27,62,63]. Several studies, including ours, have shown that exposure to hyperglycemia decreased the gene or protein levels of GIPR in HUVECs and several types of monocytes/macrophages [21,27,63]. We have also found that *Gipr* gene expression levels are halved in the aortas and pancreases of hyperglycemic *db*/*db* mice compared with normoglycemic wild-type mice [27]. On the other hand, another report showed that *Gipr* gene expression levels were not changed in mouse aortic ECs, but upregulated in mouse aortic SMCs under hyperglycemic conditions in vitro [62]. Furthermore, *Gipr* gene expression levels were upregulated in the carotid arteries of diabetic LDL receptor-knockout mice compared with non-diabetic mice in vivo [62]. Therefore, further studies are needed to clarify whether GIPR expression in vascular cells can be altered in diabetic conditions. However, given the fact that GIP suppressed the atherosclerosis and restenosis in diabetic mice [21,27], beneficial cardiovascular effects of GIP are likely to be preserved, at least partly, in diabetic conditions. 

### 5.2. GIPR Desensitization under Chronic Stimulation

GIPR undergoes very rapid and reversible homologous desensitization upon GIP binding, which is considered an important response to regulate the GIP actions in GIPR-expressing cells [119]. Thus, there is a concern that chronic administration of GIP may lead to impaired actions of GIP through GIPR desensitization. An early study showed that the continuous infusion of human GIP to normal rats increased the blood levels of insulin up to 30 min, which was gradually decreased afterward, returning to the baseline levels at 4 h [120]. Similarly, in cAMP reporter L-cells expressing rat GIP receptor, human GIP at 2 nM increased the cAMP-dependent β-galactosidase production up to 4 h, which was disappeared at 16 h after GIP stimulation. In addition, pre-incubation with GIP at 2 nM for 16 h also diminished the effects of subsequent GIP stimulation on cAMP-dependent β-galactosidase production. Another study also reported the desensitization of GIPR after GIP stimulation in cultured differentiated mouse 3T3-L1 adipocytes [121]. GIP stimulation at 100 nM for 60 min increased the cAMP levels, which was approximately halved in the second GIP stimulation at 100 nM for 15minutes in association with decreased numbers of GIPR on the cell surface after the first GIP stimulation.

There are a couple of studies demonstrating that insulinotropic effects of GIP can be preserved after chronic stimulation [122,123]. Degradation resistant GIP analog, N-AcGIP (LysPAL [37]) at 12.5 nmol/kg/day once daily was injected to diabetic *ob/ob* mice, but its insulinotropic effects were similarly observed at 14 days after the serial injections [122]. In addition, another study reported that insulinotropic effects of GIP were not impaired by exposure to slightly supra-physiological concentrations of GIP in healthy subjects and age- gender- and weight-matched patients with type 2 diabetes and first-degree relatives of such patients [123]. GIP at 50 pmol/kg was injected to the subjects before and after continuous GIP infusion at 2 pmol/kg/min for 150 min. However, there was no difference between insulinotropic effects of the first and second GIP injections in any of the groups. These findings suggest that GIP-induced cAMP production may be blunted to some extent after chronic GIP stimulation via GIPR desensitization, but it can be still sufficient to stimulate insulin secretion in pancreatic beta cells. 

## 6. Further Perspective: Potential Effects of GIP as an Enhancer for GLP-1 Actions

Recent in vitro studies have demonstrated that GIP may enhance GLP-1 actions through binding to GLP-1R/GIPR heterodimer. GIPR and GLP-1 belong to G protein-coupled receptors, which function as not only monomers but also heterodimers or homodimers [124,125]. Several studies reported that GLP-1R/GIPR heterodimer was formed in HEK-293 cells expressing these receptors, and the heterodimer resulted in an impaired response to GLP-1 stimulation [126,127,128]. Interestingly, the heterodimer formation was promoted by GLP-1 stimulation, whereas it was reversed by GIP stimulation [127]. These findings suggest a possibility that GIP can act as an enhancer of GLP-1 actions in cells expressing both GLP-1R and GIPR such as VECs. Although these in vitro findings need to be confirmed by in vivo studies, this effect of GIP may be one of mechanisms, by which the GLP-1R/GIPR dual agonist LY3298176 exerted superior metabolic effects compared with the GLP-1R mono-agonist dulaglutide [54].

## 7. Conclusions

**Figure 1:**
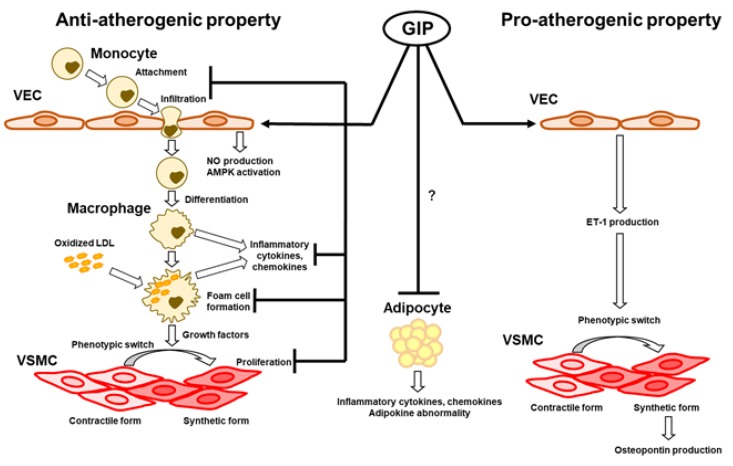
Anti-atherogenic and proatherogenic properties of GIP in vasculatures.

GIP can exhibit both anti-atherogenic and pro-atherogenic properties in vitro: the former is enhancement of NO production and AMPK activation in VECs, inhibition of cell proliferation in VSMCs, and suppression of inflammatory responses in monocytes, macrophages, and adipocytes. The latter is enhancement of ET-1 production in VECs and osteopontin production in VSMCs, and provocation of inflammatory responses in adipocytes (Figure 1). However, overall effects of GIP at pharmacological concentrations are likely to be protective against atherosclerosis both in non-diabetic and diabetic conditions in vivo. Furthermore, recent in vivo studies have demonstrated multiple beneficial effects of GIP on diabetes-related diseases, such as Alzheimer’s disease [129,130,131,132,133,134] and osteoporosis [135,136,137,138,139,140,141,142,143,144,145,146]. These findings suggest that dual or triple agonists including GIPR, which will be available in the near future [54,55,56], could be comprehensive treatment for diabetes and its related disorders. 

Anti-atherogenic effects of GIP are induced by enhancement of nitric oxide (NO) production and AMP-activated protein kinase (AMPK) activation in vascular endothelial cells (VECs), suppression of cell proliferation in vascular smooth muscle cells (VSMCs), and suppression of inflammatory responses and form cell formation in monocytes/macrophages or adipocytes. Pro-atherogenic effects of GIP are associated with enhancement of endothelin-1 (ET-1) production in VECs, ET-1-mediated osteopontin production in VSMCs, and provocation of inflammatory responses in adipocytes.

## Figures and Tables

**Table 1:** Effects of glucose-dependent insulinotropic polypeptide (GIP) on cultured cells related to cardiovascular disease. ↑, increase; →, no change; ↓ decrease.

Cell Type		Anti-atherogenic	Pro-atherogenic
VEC	Canine portal vein EC	↑ NO production [64]	
	Canine hepatic artery EC		↑ ET-1 level [64]
	HUVEC	↓ AGEs-induced oxidative stress and inflammation [61] ↑ NO production [27,63] ↑ AMPK activation [27] ↓ iNOS level [63]	↑ ET-1 level [60]
	Mouse aortic EC		↑ ET-1 level [62]
VSMC	Human aortic SMC	↓Growth factor-induced cell proliferation [20]	
	Mouse aortic SMC		→ Osteopontin level [62]
Monocyte /macrophage	Human THP-1 cell	↓ Inflammation [76] ↓ Migration [77]	
	Mouse RAW 264 cell	↓ Inflammation [77] ↓ Migration [77]	↑ Inflammation [78]
Adipocyte	Isolated human adipocyte	↑ Adiponectin level [79] Inflammation [80]	
	Isolated rat adipocyte	↑ Adiponectin level [79]	↑ Inflammation [81] ↑ Osteopontin level [82,83]
	Mouse 3T3-L1 cell		↑ Inflammation [82,84] ↑ Osteopontin level [83] → Inflammation [78] ↓ Adiponectin level [84]

**Table 2:** Cardiovascular effects of GIP in animal models of cardiovascular disease. ↑, increase; →, no change; ↓ decrease.

Animal Model		GIPR Activation	GIPR Inhibition
Atherosclerosis	ApoE knockout (male C57BL/6-background mice)	↓Plaque formation [19] ↓Macrophage foam cell formation [19] ↑Plaque stability [77]	
	ApoE knockout with diabetes (male C57BL/6-background mice)	↓Plaque formation [20] ↓Macrophage foam cell formation [20]	
Restenosis	Femoral artery wire injury (male C57BL/6 mice)	↓Neointimal formation [27] ↑endothelial regeneration [27]	↑Neointimal formation [27]
	Femoral artery wire injury with diabetes (male *db/db* mice)	↓Neointimal formation [27]	
Cardiac remodeling	Angiotensin II infusion (male C57BL/6-background ApoE knockout mice)	↓Cardiomyocyte enlargement [25] ↓interstitial fibrosis [25]	
	Coronary artery ligation (male C57BL/6-background mice)	↑Scar formation [99]	↓Mortality [99] ↓Scar formation [99]
	Transverse aortic constriction (male C57BL/6-background mice)		→Left ventricular function [99]
	Doxorubicin injection (male C57BL/6-background mice)		↓Cardiac atrophy [99] → Mortality [99]
Inflammation	Standard diet (Ref. 49: male C57BL/6 mice, Ref. 86: male *db mysty* mice)	↓Adipose tissue inflammation [49] ↑Adipose tissue inflammation [82]	
	High fat diet (Ref. 50: C57BL/6-background mice [no information for sex], Ref. 89: male F344/jcl rats, Ref. 90: male C57BL/6 mice, Ref. 109: no information for background strain and sex)	↓Adipose tissue inflammation [50,79,80] ↑Adipose tissue expression and blood levels of adiponectin [79,80]	↑Blood and adipose tissue levels of IL-6 [100]
	Diabetes (male *db/db* mice)	↑Adipose tissue inflammation [82]	
	Gingivitis (male C57BL/6-background mice)		↑Gingival inflammation [76]
	Endotoxemia (male C57BL/6 mice)	↓ Blood IL-6 level [101]	

**Table 3:** Cardiovascular effects of GIP in human studies. ↑, increase;↓ decrease.

Subject	Change
Normal glucose tolerance or young healthy	↓Mean arterial blood pressure [114] ↑Heart rate [114] ↑Femoral artery blood flow [115] ↑ Blood levels of CCL2 [78], CCL8 [78], and osteopontin [62] ↑Adipose tissue levels of *CCL2* [78], *CCL8* [78], and *IL-6* [78]
Impaired glucose tolerance	↓Mean arterial blood pressure [114] ↑Heart rate [114]
Type 2 diabetes	↓Mean arterial blood pressure [114] ↑Heart rate [114]

## Abbreviations

AGE	Advanced glycation end product
AMPK	AMP-activated protein kinase
ApoE-/-	Apolipoprotein E knockout
CaMKK	Calcium/calmodulin-dependent protein kinase kinase
cAMP	Cyclic adenosine monophosphate
CCL	Chemokine ligand
CREB	cAMP response element-binding protein
CVD	Cardiovascular disease
DPP	Dipeptidyl peptidase
ET	Endothelin
GIP	Glucose-dependent insulinotropic polypeptide
GIPR	GIP receptor
GLP-1	Glucagon like peptide-1
GLP-1RA	GLP-1 receptor agonist
HUV	Human umbilical vein
IL	Interleukin
LDL	Low density lipoprotein
LKB	Liver kinase B
LPS	Lipopolysaccharide
NO	Nitric oxide
NOS	Nitric oxide synthase
PAI	Plasminogen activator inhibitor
PKA	Protein kinase A
PLC	Phospholipase C
PTA	Percutaneous transluminal angioplasty
SMC	Smooth muscle cell
TNF	Tumor necrosis factor
VEC	Vascular endothelial cell
VSMC	Vascular smooth muscle cell
